# Comparison of Risk Factors for Habitual Substance Use Among Adolescents in Korea by Maternal Nationality: Analysis of 18th and 19th Korea Youth Risk Behavior Surveys (2022 and 2023)

**DOI:** 10.3390/children12040458

**Published:** 2025-04-03

**Authors:** Hyeon Ok Ju, So Yeon Park

**Affiliations:** College of Nursing, Dong-A University, Busan 49201, Republic of Korea

**Keywords:** adolescent, cultural diversity, substance abuse, oral, health risk behaviors

## Abstract

**Background/Objectives**: This study identified the risk of habitual substance use among multicultural adolescents, focusing on health behavior characteristics and cultural differences based on their mother’s country of origin. **Methods**: This secondary data analysis used data from the 18th (2022) and 19th (2023) Korea Youth Risk Behavior Web-based Surveys, which are repeated cross-sectional surveys conducted annually. The analyses of 82,520 adolescents included descriptive statistics, the Rao–Scott χ^2^ test, and logistic regression. **Results**: The prevalence of habitual substance use among multicultural adolescents whose mothers were from the examined countries was 2.01%, which is 0.74 percentage points higher than the 1.27% prevalence observed among adolescents with Korean mothers. Among multicultural adolescents, the risk of habitual substance use was higher under specific conditions: those with Chinese mothers faced increased risks when not living with family (OR = 6.22) or smoking (OR = 12.65); Korean-Chinese adolescents had higher risks when experiencing suicidal ideation (OR = 3.41) or anxiety (OR = 8.17); and those with Vietnamese mothers were at greater risk when exposed to violence (OR = 12.42) or depression (OR = 14.06). These results underscore the role of cultural and psychological factors in adolescent substance use. **Conclusions**: Our findings revealed differences in risk factors based on the mother’s country of origin. These results underscore the importance of understanding adolescents’ unique characteristics and developing tailored intervention strategies that account for these cultural and familial differences.

## 1. Introduction

Substance abuse is a major global public health concern. Data from 2022 indicate that approximately 1 in every 18 individuals globally will experience substance abuse, with an alarming annual increase of nearly 20% [1]. In particular, the use of substances outside the scope of medical monitoring significantly increases the risk of health problems [2]. Moreover, the accessibility of these substances has been facilitated by evolving environments where they can be increasingly obtained through online platforms or messaging services via various channels [3]. The accessibility of medications has increased in South Korea since 2012, when the over-the-counter sale of nonprescription drugs outside pharmacies was permitted. Following the COVID-19 pandemic, the consumption of analgesics and cold medications has further increased [4]. According to a 2023 survey of emergency medical facilities in South Korea [5], intoxication caused by therapeutic medications accounted for 50.8% of all intoxication cases. Among adolescents, this rate was significantly higher, with 80.5% of the intoxication cases linked to therapeutic medications, particularly analgesics.

Adolescence is a critical period relating to the initiation of substance use [2]. Caregivers often assume that adolescents have a sufficient understanding of medication and therefore delegate decisions about its use [6,7]. Many adolescents progress from initial therapeutic use to habitual use and addiction. Risk factors for habitual substance use include anxiety, loneliness, not living with parents, and stress [8,9,10]. As a transitional stage from childhood to adulthood, adolescence involves achieving developmental milestones within the family, school, and society while undergoing rapid physical and psychological changes. This period is also associated with risky behaviors, such as smoking, alcohol consumption, sexual risk-taking, violence, and substance use [11]. Substance use not only poses personal challenges but also contributes to significant social and economic burdens. In particular, habitual substance use during adolescence has been associated with serious negative consequences for psychological well-being, including reduced life satisfaction, lower self-rated health, and increased risks of depression and anxiety [12]. Parental influence, especially the presence of a mother, plays a crucial role in mitigating these risks. The mere presence of a mother strengthens an adolescent’s ability to make safe decisions in risky social situations [13]. When faced with stress, a maternal presence helps adolescents regulate excessive worry, anxiety, and avoidance, offering them greater acceptance and reassurance. A flexible and supportive mother–child relationship provides a secure base, fostering secure attachment, promoting emotional resilience, and offering a sense of stability to explore the unknown [14]. This bond serves as an effective buffer against adolescent anxiety and risk behaviors [15].

In South Korea, the number of multicultural families has been increasing annually. According to 2023 national vital statistics [16], the number of multicultural marriages rose by 17.2% compared with the previous year, with 69.8% of these families consisting of foreign mothers and Korean fathers. Chinese nationals represent the largest proportion of married migrant women, while the number of Vietnamese nationals is rapidly increasing. The number of children from multicultural families has been consistently rising, accounting for 3.47% of the total student population by 2023 [16]. In this study, maternal nationalities were categorized based on the frequency of occurrence reported in the 2023 national vital statistics [16]. The most prevalent groups—Vietnamese, Korean-Chinese, and Chinese—were selected for analysis alongside Korean mothers. These categories reflect the dominant multicultural family compositions in Korea and were selected to support analytical validity in group comparisons.

Despite the growing cultural diversity within South Korea and the fact that a significant proportion of adolescents from multicultural families are born and raised in the country, many still face difficulties adapting to their environment [17]. Married migrant women often experience substantial stress due to challenges in cultural adaptation, a lack of social support, conflicts with spouses, and difficulties in raising children, with a high prevalence of depression reported in this population [18]. The severity of these challenges varies with the cultural differences between the country of origin and Korea [19]. However, these differences remain underexplored in the research and intervention strategies, highlighting the need for targeted studies and practical applications. Research on the mental health and health-risk behaviors of multicultural adolescents in Korea has shown differences based on the mother’s country of origin [20].

Adolescents from multicultural families are often influenced by their mothers’ stress and anxiety, which can heighten their own stress levels and lead to health-risk behaviors [21]. They are particularly vulnerable to addictions such as internet use, gaming, gambling, and substance abuse [22], with a high risk of habitual substance use [9,23]. As multicultural families become more common in Korean society, addressing substance use in this group is an emerging concern. Understanding the risk factors associated with habitual substance use—especially those influenced by maternal nationality—can provide essential data for prevention and intervention. Such insights are critical for developing tailored programs that support the well-being and social integration of multicultural adolescents. Given the limitations of secondary data, a comprehensive theoretical framework could not be fully applied. However, this study is grounded in previous research on adolescent risk behaviors and multicultural adaptation, and it incorporates relevant conceptual insights to interpret the findings.

This study aims to examine the risk levels of habitual substance use among multicultural adolescents, with a focus on health behavior characteristics and cultural differences based on the mother’s country of origin. The specific objectives of this study are to compare substance use among multicultural adolescents based on their health behavior characteristics and to identify factors influencing substance use in both multicultural and non-multicultural adolescents.

## 2. Materials and Methods

### 2.1. Study Design

To identify the predictive factors influencing substance use among adolescents, we performed a secondary data analysis using raw data from the 18th and 19th Korea Youth Risk Behavior Web-Based Surveys (KYRBS).

### 2.2. Participants and Data Collection

The KYRBS is an anonymous, self-administered online survey conducted annually to assess the health behaviors of Korean adolescents, including smoking, drinking, obesity, dietary habits, and physical activity. The survey targeted middle and high school students (grades 7–12) in South Korea. To minimize sampling errors, the population was stratified into 39 regional groups and school levels. Proportional allocation methods were applied based on the urban area, school level, and school type, and participants were selected using stratified cluster sampling. The 18th KYRBS was conducted between 29 August and 25 October 2022, with 51,850 students participating from 798 middle and high schools in 17 provinces across the country, achieving a participation rate of 92.2%. The 19th KYRBS was conducted from 28 August to 19 October 2023 and involved 52,880 students from 799 middle and high schools, with a participation rate of 92.9%. The KYRBS data were collected using unique, nonidentifiable codes, ensuring anonymity and confidentiality without collecting personal information.

For this study, we utilized data from 2022 and 2023, as this period marks the time when all students had fully resumed in-person schooling after the COVID-19 pandemic. The South Korean government downgraded the COVID-19 alert level in April 2022, ensuring a stable educational environment for all students. Additionally, the KYRBS is designed to prevent duplicate participation across survey cycles, maintaining the integrity of the dataset and minimizing potential bias. Multicultural and non-multicultural adolescents were classified based on the survey question, “Was your mother born in Korea?” The response “No” indicated multicultural backgrounds. Among the multicultural participants, students were further categorized based on their responses to the question, “In which country was your mother born?” This study focused on students whose mothers were born in China (Korean-Chinese), China (Han or other ethnic groups), or Vietnam. For analytical purposes, we categorized China (Korean-Chinese) as “Korean-Chinese” and China (Han or other ethnic groups) as “Chinese”.

### 2.3. Methods

The 18th KRYBS [24] included 114 questions and 101 indicators, whereas the 19th Survey [25] comprised 88 questions and 92 indicators. In this study, substance use for non-therapeutic purposes was set as the dependent variable, and the explanatory variables were selected based on previous research [26].

#### 2.3.1. General Characteristics

The general characteristics included in this study were sex, school type, socioeconomic status, and cohabitation with family members. Socioeconomic status was reclassified into three categories: “High” (upper and upper-middle), “Middle”, and “Low” (lower-middle and lower). For the question, “What is your current living arrangement?” participants who selected options such as living with relatives, in boarding houses, on their own, in dormitories, or in childcare facilities were reclassified as “Not living with family”, while those living with their family were categorized as “Living with family”.

Habitual substance use was categorized based on self-reported responses in the KYRBS. Participants were asked the following survey question: “Have you ever habitually or intentionally used drugs (such as tranquilizers, stimulants, sleeping pills, appetite suppressants, or opioid analgesics) or inhaled substances such as glue (adhesives), cannabis, cocaine, or butane gas?” Respondents who answered “Yes” were classified into the habitual substance use group. This definition ensures that only those engaging in intentional or repeated non-therapeutic substance use are included, distinguishing them from occasional or unintentional users. It also reflects the survey developers’ intent to capture patterns of habitual or intentional misuse rather than one-time experimentation.

Habitual substance use was categorized based on self-reported responses in the KYRBS. Participants were asked the following survey question: “Have you ever habitually or intentionally used drugs (such as tranquilizers, stimulants, sleeping pills, appetite suppressants, or opioid analgesics) or inhaled substances such as glue (adhesives), cannabis, cocaine, or butane gas?” Respondents who answered “Yes” were classified into the habitual substance use group. This definition ensures that only those engaging in intentional or repeated non-therapeutic substance use are included, distinguishing them from occasional or unintentional users. It also reflects the survey developers’ intent to capture patterns of habitual or intentional misuse rather than one-time experimentation.

#### 2.3.2. Health Behavior and Mental Health Characteristics

Health behavior includes perceived health status, sleep satisfaction, current drinking, and current smoking status. Perceived health status was reclassified into three categories: “Healthy” for responses of “Very healthy” or “Healthy”, “Fair” for “Average”, and “Unhealthy” for “Unhealthy” or “Very unhealthy”. Sleep satisfaction was reclassified as “Yes” if participants reported their recent sleep duration as “Very sufficient” or “Sufficient” for relieving fatigue; other responses were categorized as “No”. Alcohol consumption and smoking were assessed based on experiences excluding traditional rituals, categorizing participants based on their current usage status.

Mental health includes psychological well-being and related factors, such as school violence, stress, depression, suicidal ideation, suicide planning, loneliness, and anxiety. School violence was assessed in relation to exposure to or experience of violent incidents. Stress perception was categorized as “Yes” for responses of “Very much” or “A lot”, and “No” for other responses. Depression, suicidal ideation, suicide planning, and suicide attempts were classified based on responses indicating the presence or absence of these experiences within the past 12 months. Loneliness was categorized as “Yes” if participants reported feeling “Always” or “Often” lonely and “No” for other responses. Anxiety was evaluated using a modified version of Spitzer’s original tool [27], with a 4-point scale adjusted to a 0–3 point scale. Anxiety scores were reclassified as follows: scores below 5 were categorized as “None”, 5–9 as “Mild”, 10–14 as “Moderate”, and 15–21 as “Severe”.

### 2.4. Ethical Considerations

This study adhered to the Ethical Guidelines for Research Involving Human Subjects. Raw data were obtained from the Korea Disease Control and Prevention Agency following the submission and approval of a data access request in accordance with the agency’s regulations for data utilization. The data were anonymized and de-identified to ensure that none of the participants’ personal information was accessible. Furthermore, the study received confirmation of exemption from review by the Institutional Review Board of D University as it involved a secondary analysis of publicly available, anonymized data.

### 2.5. Data Analysis

We used a complex sample design for the data analysis. The design plan file provided by the Korea Youth Risk Behavior Survey was used to incorporate information on stratification, clustering, weighting, and finite population correction factors. The data analysis was conducted using IBM SPSS Statistics 26 software. A descriptive statistical analysis was used to analyze the participants’ general characteristics, problem behaviors, and health behaviors, which were presented as frequencies that did not reflect the weights and as percentages that reflected the weights. Substance use based on participants’ general characteristics, problematic behaviors, and health behavior characteristics was analyzed using the complex samples general linear model and the Rao–Scott χ^2^ test for complex sample cross-tabulation. The Rao–Scott test was chosen over the Pearson chi-square test because, for data obtained from complex survey designs, it provides more stable and reliable statistical estimates. Factors influencing substance use were analyzed using complex sample multiple logistic regression. Independent variables that showed statistical significance in the univariate analysis (*p* < 0.05) were included in the model. Odds ratios (ORs) and 95% confidence intervals (CIs) were calculated for each variable. To identify risk factors for habitual substance use across cultural backgrounds, we conducted separate logistic regression analyses for each subgroup of adolescents based on maternal nationality (Chinese, Korean-Chinese, and Vietnamese). In each model, adolescents with Korean-born mothers served as the reference group. This approach allowed for the identification of unique risk factors within each multicultural subgroup. While multiple variables were included in each model based on statistical significance from the univariate analysis, we acknowledge the potential risk of overadjustment bias. This approach was intended to identify concurrent influences of multiple risk factors within each subgroup.

## 3. Results

### 3.1. General and Health Behavior-Related Characteristics by Maternal Country of Origin

The prevalence of habitual substance use among multicultural adolescents whose mothers were from the examined countries was 2.01%, while a prevalence of 1.27% was observed among adolescents with Korean mothers. The general and health behavior-related characteristics of the adolescents varied significantly based on their maternal nationality and substance use experience (Table 1). In particular, adolescents from lower socioeconomic backgrounds had higher substance use rates, which were statistically significant among those with Chinese, Korean-Chinese, and South Korean mothers. Differences in cohabitation with family were also significant for those with mothers from China and South Korea. Moreover, adolescents who experienced school violence, alcohol consumption, smoking, or mental health issues (stress, depression, suicidal ideation and planning, and anxiety) were more likely to engage in substance use.

### 3.2. Factors Influencing Substance Use Experience by Maternal Country of Origin

The logistic regression analysis revealed that the key factors influencing habitual substance use included school violence experience, depression, anxiety, smoking, and alcohol consumption, although the strength and significance of these associations varied by maternal country of origin (Table 2).

#### 3.2.1. Mothers from China

Among adolescents whose mothers were from China, not living with family was strongly associated with habitual substance use, with odds 6.22 times higher than those for adolescents living with family. Similarly, adolescents who perceived their health as poor had 6.71 times higher odds of substance use compared to those with good perceived health. Smoking and alcohol use were also powerful predictors, indicating a markedly elevated risk. In contrast, perceived stress and depression were not significantly associated, while moderate anxiety showed a meaningful relationship with increased odds.

#### 3.2.2. Mothers from Korea–China

In the Korean-Chinese maternal group, moderate anxiety was significantly associated with habitual substance use, with odds 8.17 times higher (95% CI = 1.03–64.99) than those for adolescents with no anxiety. Severe anxiety and suicidal ideation also increased the likelihood of substance use, though other factors such as smoking, alcohol use, and perceived health status were not statistically significant in this group.

#### 3.2.3. Mothers from Vietnam

Among adolescents whose mothers were from Vietnam, depressive symptoms were a particularly strong predictor of habitual substance use, with adolescents having depression showing 14.06 times higher odds compared to those without. Additionally, experience of school violence was significantly associated with substance use, with odds more than 12.42 times higher. Poor perceived health and loneliness were not significant in this group.

#### 3.2.4. Mothers from South Korea

In adolescents with Korean-born mothers, a broader range of risk factors was associated with habitual substance use. Not living with family was linked to 6.22 times higher odds, and poor perceived health to 1.49 times higher odds. Smoking, alcohol use, and exposure to school violence were also significant predictors. Furthermore, depression, anxiety, suicidal ideation, and suicidal plans all showed meaningful associations with increased risk.

## 4. Discussion

This study aimed to identify the risk factors associated with habitual substance use among adolescents from multicultural families using data from the 2022 and 2023 Korean Youth Risk Behavior Surveys, the most recent nationwide datasets on adolescent health in South Korea. These datasets provide valuable insights into current trends, particularly in a post-COVID-19 context where students have returned to stable in-person learning.

In 2022, the survey questionnaire was revised to clarify the definition of habitual substance use by explicitly excluding substances such as coffee, energy drinks, and vitamins, reducing potential misinterpretations. This refinement ensures a more accurate identification of adolescents who intentionally or habitually engage in non-therapeutic drug or substance use. As the most up-to-date and methodologically refined data available, these findings offer a robust representation of current adolescent substance use behaviors, supporting the development of targeted prevention and intervention strategies. This study focused on analyzing differences by the mother’s country of origin.

South Korea is rapidly transitioning into a multicultural society. Despite longstanding concerns regarding the challenges faced by children from multicultural families in integrating into Korean society, substantive efforts to address these issues remain limited [28,29]. The prevalence of habitual substance use among multicultural adolescents whose mothers were from the examined countries was notably higher than that among adolescents with Korean mothers. In this study, survey weights were applied in the analysis based on the complex sample design. The wider confidence intervals observed in the results may be a consequence of this. Nevertheless, the overall findings and associations remained consistent, supporting the validity of the study.

Parental influence varies significantly across cultures, and applying a universal framework to understand adolescent risk behaviors may fail to account for culturally specific dynamics. Research suggests that maternal–infant interactions differ across cultures in terms of emotional expression and affective engagement, highlighting the importance of culturally tailored approaches in parenting and adolescent development [30]. Therefore, interventions targeting adolescent risk behaviors should incorporate cultural differences to enhance their effectiveness.

Chinese-origin adolescents demonstrated higher substance use rates when living apart from family, reporting poor health, consuming alcohol, smoking, or having a history of suicide planning. In China, Confucian values have historically emphasized nationalism and familism, although these values have weakened due to the transition toward nuclear family structures [27]. Nevertheless, the family unit continues to serve as a stabilizing protective factor in Chinese society [31]. Cultural characteristics often act as protective factors against high-risk behaviors. In this study, the elevated risk of habitual substance use among adolescents not cohabiting with their families can be interpreted within this cultural context. Zhang et al. [32] reported that the self-medication rate in China has reached 99.06%, which is attributed to policies such as the Chinese medical insurance system. Adolescents and individuals with limited health insurance knowledge often self-administer medications based on their perceived health condition or drug efficacy. This aligns with the findings of the present study in which subjective perceptions of health status emerged as significant risk factors. In the Chinese context, alcohol consumption and smoking are often regarded as social facilitators [33], and adolescents tend to perceive these behaviors less seriously than others. The identification of alcohol consumption and smoking as risk factors underscores the potential progression toward “tri-use” (the concurrent use of alcohol, smoking, and substances), consistent with previous research [34].

Suicidal ideation and anxiety were significant risk factors in adolescents with mothers of Chinese-Korean origin (Joseonjok). Discrimination and stigmatization against Chinese-Korean communities have intensified post-COVID-19, particularly in schools, where overt acts of prejudice and hate have been reported [34]. The media frequently depicts Chinese Koreans as criminals, perpetuating negative stereotypes [35,36]. Such discrimination adversely impacts maternal satisfaction with life in South Korea, hinders parent–child relationships, and negatively influences adolescent development [37]. Studies on ethnic discrimination [22] indicate that Asian adolescents exhibit increased substance use, which correlates with suicide risk, despite not showing significant increases in alcohol or tobacco consumption. This aligns with the findings of the present study, highlighting the necessity of addressing the stigma and discrimination faced by Chinese-Korean adolescents. Lee [38] also reported that suicidal ideation was associated with an elevated risk of habitual substance use among adolescents, with its severity exacerbated by stress or anxiety. This is consistent with the findings of the present study that identified suicidal ideation and anxiety as risk factors for habitual substance use among Chinese-Korean adolescents.

For adolescents with mothers of Vietnamese origin, school violence and depression were identified as risk factors for substance use. School violence is closely associated with adolescent depression [39], highlighting the need for targeted approaches to address depression among adolescents with mothers from Vietnam. A prior study noted that Vietnamese immigrant women often face challenges due to their low socioeconomic status, language barriers, cultural differences, and variations in educational practices [40]. Maternal acculturation stress diminishes parenting efficacy, which is directly related to the cultural adaptation stress experienced by multicultural adolescents [17,37,40]. Adolescent cultural adaptation stress significantly affects depression during adolescence [41], which is also associated with substance use and is consistent with the findings of this study. Additionally, previous studies analyzing risk behaviors and mental health determinants among Vietnamese adolescents have indicated that urban adolescents are more likely to engage in high-risk behaviors. Factors such as mother–child bonding, exposure to violence, and suicidal ideation were significantly associated with adolescent risk behaviors such as drug use [42]. These findings support the results of the present study, reinforcing the critical role of maternal relationships and exposure to adverse experiences in shaping adolescent substance use. Furthermore, research on Vietnamese immigrant families suggests that despite adapting to a new environment, immigrant parents often retain cultural values from their country of origin, leading to intergenerational conflicts within the family. Such conflicts may, in turn, manifest as risk behaviors among adolescents [43]. Choi et al. [43] emphasized the necessity of understanding the cultural background of the country of origin when addressing issues in multicultural families, asserting that culturally tailored approaches are essential for the effective prevention of adolescent risk behaviors. The large effect sizes observed in this study highlight the serious impact of these behavioral and psychosocial factors. These results suggest that habitual substance use is shaped not only by individual characteristics but also by broader cultural and social influences related to maternal nationality.

The risk factors identified in this study align with prior research on habitual substance use among Korean adolescents [8,9,10]. The present study extends this analysis by incorporating maternal nationality as a comparative framework. Adolescents navigating dual cultural contexts often face challenges such as academic underachievement, interpersonal difficulties, bullying, and school maladjustment due to differences in physical appearance, which can lead to mental health issues such as depression, anxiety, and suicidal behavior, as well as maladaptive behaviors such as smoking, alcohol consumption, violence, and substance use [44,45]. Addressing habitual substance use during adolescence is critical as it can result in long-term health problems in adulthood. Broad approaches focusing solely on multicultural adolescents may overlook their distinct needs, underscoring the need for culturally sensitive and individualized health interventions to effectively mitigate these risks. Therefore, intervention programs should move beyond general multicultural classifications and incorporate culturally specific factors. Policies aimed at early prevention should prioritize high-risk groups based on maternal background and provide tailored support systems within schools and communities.

### Limitations

This study had several limitations. First, although we used large-scale data from the KYRBS, its cross-sectional design limits the ability to establish causal relationships. Future longitudinal studies are needed to explore the pathways of habitual substance use in Korea. Second, while the KYRBS is a nationally representative survey with standardized methodologies, it does not utilize detailed psychological assessment tools. The questionnaire is designed for public health surveillance rather than in-depth psychological evaluation. However, its items have been refined over time to enhance their validity and reliability in large-scale epidemiological research. Third, the study relied on self-reported data, which may introduce recall and social desirability biases, potentially affecting the accuracy of responses regarding substance use behaviors. Future research should consider validating self-reported data with objective measures. Fourth, this study did not account for paternal influences, which may significantly impact adolescent substance use behaviors. A more comprehensive approach that includes familial and social factors would provide deeper insights into the sociocultural context of multicultural adolescents. Fifth, while multivariate logistic regression models were constructed using variables that showed statistical significance in univariate analyses, this approach may be subject to overadjustment bias. Including multiple interrelated predictors in a single model can reduce the estimate precision and potentially obscure meaningful associations. Although our intent was to explore the combined influence of relevant factors within each maternal subgroup, future research may benefit from testing each exposure separately or applying minimal adjustment models to reduce bias and enhance interpretability. Despite these limitations, the use of recent nationally representative data strengthens the relevance of the findings for future research and policy development.

## 5. Conclusions

This study used raw data from the Korea Youth Risk Behavior Survey to identify factors influencing habitual substance use among multicultural adolescents by comparing results based on maternal nationality. The findings confirmed significant differences in risk factors depending on the mother’s country of origin, with higher levels of risk observed among multicultural adolescents than among their non-multicultural counterparts in South Korea. These results underscore the need to consider maternal nationality when addressing habitual substance use among adolescents.

## Figures and Tables

**Table 1:** Relationships between sociodemographic characteristics, mental health, health behaviors, and habitual substance use among adolescents by maternal country of origin (N = 82,520).

Variable	Category	China (n = 486)			Korea–China (n = 579)			Vietnam (n = 1023)			Korea (n = 80,432)		
		Substance Use		χ^2^ (*p*)	Substance Use		χ^2^ (*p*)	Substance Use		χ^2^ (*p*)	Substance Use		χ^2^ (*p*)
		No	Yes		No	Yes		No	Yes		No	Yes	
		n (%)	n (%)		n (%)	n (%)		n (%)	n (%)		n (%)	n (%)	
Sex	Male	238 (50.1)	6 (54.0)	0.08 (0.752)	235 (41.0)	6 (47.2)	0.23 (0.660)	513 (50.4)	12 (72.3)	3.27 (0.085)	38,153 (48.9)	411 (40.8)	26.84 (<0.001)
	Female	236 (49.9)	6 (46.0)		331 (59.0)	7 (52.8)		493 (49.6)	5 (27.7)		41,253 (51.1)	615 (59.2)	
Economic status	High	131 (27.2)	3 (22.0)	25.33 (<0.001)	156 (26.8)	6 (43.1)	12.55 (0.003)	189 (17.7)	4 (14.1)	2.72 (0.298)	34,923 (44.9)	448 (45.6)	84.44 (<0.001)
	Medium	254 (53.1)	5 (37.9)		314 (55.8)	2 (12.1)		549 (54.5)	6 (41.1)		35,989 (44.8)	376 (36.0)	
	Low	89 (19.7)	4 (40.1)		97 (17.4)	5 (44.8)		268 (27.8)	7 (44.8)		8494 (10.2)	202 (18.4)	
Residential type	Family living	456 (97.0)	10 (83.7)	6.85 (0.007)	535 (94.7)	11 (90.9)	0.42 (0.445)	941 (93.8)	15 (95.6)	0.09 (0.655)	76,317 (96.7)	947 (92.8)	48.00 (<0.001)
	Non-family living	18 (3.0)	2 (16.3)		31 (5.3)	2 (9.1)		65 (6.2)	2 (4.4)		3089 (3.3)	79 (7.2)	
Perceived health status	Healthy	269 (57.6)	3 (25.9)	22.78 (<0.001)	324 (57.0)	6 (47.0)	2.31 (0.364)	630 (61.4)	8 (49.9)	3.29 (0.264)	51,163 (64.3)	429 (41.6)	471.99 (<0.001)
	Fair	136 (29.0)	2 (13.4)		160 (28.3)	3 (23.9)		277 (28.2)	4 (26.3)		20,302 (25.6)	295 (28.8)	
	Unhealthy	69 (13.4)	7 (60.8)		82 (14.8)	4 (29.1)		99 (10.3)	5 (23.8)		7941 (10.1)	302 (29.7)	
Sleep satisfaction	No	131 (28.1)	4 (27.1)	0.01 (0.925)	124 (21.6)	3 (25.0)	0.10 (0.740)	335 (32.2)	2 (9.3)	4.09 (0.066)	19,380 (24.1)	141 (13.8)	60.14 (<0.001)
	Yes	343 (71.9)	8 (72.9)		442 (78.4)	10 (75.0)		671 (67.8)	15 (90.7)		60,026 (75.9)	885 (86.2)	
Current drinking	No	318 (66.4)	4 (27.8)	8.41 (<0.001)	367 (64.4)	7 (57.2)	0.33 (0.577)	729 (72.5)	6 (43.2)	7.25 (0.017)	53,970 (68.0)	400 (39.3)	390.20 (<0.001)
	Yes	156 (33.6)	8 (72.2)		119 (96.9)	6 (42.8)		277 (27.5)	11 (56.8)		25,436 (32.0)	626 (60.7)	
Current smoking	No	447 (94.0)	7 (59.2)	23.76 (<0.001)	502 (89.0)	10 (79.0)	1.46 (0.154)	937 (93.1)	12 (74.6)	8.68 (0.011)	73,401 (92.4)	718 (69.1)	778.91 (<0.001)
	Yes	27 (6.0)	5 (40.8)		64 (11.0)	3 (21.0)		69 (6.9)	5 (25.4)		6005 (7.6)	308 (30.9)	
Treatment for violence	No	462 (97.7)	9 (69.8)	33.81 (<0.001)	555 (98.1)	11 (90.9)	3.70 (0.032)	987 (98.0)	11 (67.9)	63.52 (<0.001)	77,986 (98.3)	900 (87.4)	653.28 (<0.001)
	Yes	12 (2.3)	3 (30.2)		11 (1.9)	2 (9.1)		19 (2.0)	6 (32.1)		1420 (1.7)	126 (12.6)	
Perceived stress	No	291 (63.7)	3 (22.3)	9.34 (0.002)	327 (58.5)	6 (47.4)	0.74 (0.421)	644 (63.2)	5 (28.4)	8.79 (0.005)	48,559 (61.1)	318 (30.8)	398.67 (<0.001)
	Yes	183(36.3)	9 (77.7)		239 (41.5)	7 (52.6)		362 (36.8)	12 (71.6)		30,847 (38.9)	708 (69.2)	
Depression	No	338 (72.4)	6 (47.7)	3.80 (0.031)	390 (68.4)	4 (37.2)	6.47 (0.023)	746 (73.2)	3 (9.2)	34.51 (<0.001)	58,375 (73.6)	380 (37.0)	706.79 (<0.001)
	Yes	136 (27.6)	6 (52.3)		176 (31.6)	9 (62.8)		260 (26.8)	14 (90.8)		21,031 (26.4)	646 (63.0)	
Suicidal ideation	No	399 (85.6)	8 (65.6)	3.99 (0.045)	451 (79.0)	5 (43.9)	10.51 (0.004)	875 (86.5)	8 (43.2)	26.15 (<0.001)	68,845 (86.7)	513 (49.5)	1203.60 (<0.001)
	Yes	75 (14.4)	4 (34.4)		115 (21.0)	8 (56.1)		131 (13.5)	9 (56.8)		10,561 (13.3)	513 (50.5)	
Suicidal plan	No	447 (94.7)	10 (78.9)	5.89 (0.029)	521 (91.8)	8 (74.3)	5.67 (0.011)	968 (96.1)	11 (62.1)	45.47 (<0.001)	75,941 (95.7)	712 (68.7)	1674.69 (<0.001)
	Yes	27 (5.3)	2 (21.1)		45 (8.2)	5 (25.7)		38 (3.9)	6 (37.9)		3465 (4.3)	314 (31.3)	
Loneliness experience	No	383 (82.1)	8 (61.7)	3.51 (0.049)	450 (80.5)	9 (77.7)	0.07 (0.785)	845 (83.6)	7 (28.0)	36.72 (<0.001)	65,444 (82.4)	532 (53.0)	602.87 (<0.001)
	Yes	91 (17.9)	4 (38.3)		116 (19.5)	4 (22.3)		161 (16.4)	10 (72.0)		13,962 (17.6)	494 (47.0)	
Generalized anxiety disorder	None	319 (68.1)	4 (27.7)	18.81 (<0.001)	349 (61.5)	6 (47.3)	22.26 (0.001)	681 (67.9)	7 (28.7)	65.04 (<0.001)	50,860 (63.8)	303 (29.8)	1183.59 (<0.001)
	Mild	89 (18.8)	2 (23.4)		123 (20.3)	0 (0.0)		197 (19.8)	1 (8.5)		18,749 (23.8)	291 (27.5)	
	Moderate	34 (6.3)	4 (34.8)		56 (10.4)	1 (11.8)		82 (8.0)	3 (17.0)		6472 (8.2)	198 (19.6)	
	Severe	32 (6.7)	2 (14.1)		38 (7.7)	6 (40.8)		46 (4.3)	6 (45.8)		3325 (4.2)	234 (23.1)	

All percentages are weighted.

**Table 2:** Factors affecting habitual substance use in adolescents by maternal country of origin (N = 82,520).

Variable	Category	China (n = 486)			Korea–China (n = 579)			Vietnam (n = 1023)			Korea (n = 80,432)		
		OR	95% CI	*p*	OR	95% CI	*p*	OR	95% CI	*p*	OR	95% CI	*p*
Economic status	High	2.09	0.73-5.99	0.135	11.69	1.38–99.12	0.052	1.61	0.29–8.72	0.854	1.36	1.14–1.52	<0.001
	Low	2.45	0.96–6.23		9.49	0.98–91.64		1.10	0.28–4.26		1.18	0.97–1.42	
	Medium (ref)												
Residential type	Non-family living	6.22	2.04–19.02	0.001	1.80	0.39–8.37	0.452	0.69	0.14–3.45	0.656	2.27	1.76–2.92	<0.001
	Family living (ref)												
Perceived health status	Healthy	0.75	0.28–1.99	<0.001	0.38	0.07–2.12	0.541	1.01	0.18–5.61	0.999	0.81	0.69–0.95	<0.001
	Unhealthy	6.71	2.34–19.29		0.56	0.07–4.62		1.00	0.16–6.33		1.49	1.25–1.79	
	Fair (ref)												
Current drinking	Yes	5.15	1.49–17.68	0.009	1.35	0.45–4.03	0.587	1.95	0.52–7.29	0.323	1.82	1.56–2.13	<0.001
	No (ref)												
Current smoking	Yes	12.65	3.40–47.08	<0.001	0.03	0.00–0.36	.007	1.62	0.34–68.04	0.800	1.62	1.19–2.21	0.002
	No (ref)												
Treatment for violence	Yes	1.74	0.41–7.36	0.449	0.38	0.03–4.78	0.452	12.42	3.23–47.82	<0.001	2.64	2.12–3.29	<0.001
	No (ref)												
Perceived stress	Yes	2.00	0.66–6.05	0.218	0.44	0.04–4.39	0.484	0.394	0.06–2.35	0.306	1.22	1.02–1.46	0.030
	No (ref)												
Depression	Yes	0.58	0.58–1.59	0.291	2.46	0.61–9.89	0.204	14.06	2.15–91.92	0.006	1.47	1.23–1.76	<0.001
	No (ref)												
Suicidal ideation	Yes	0.31	0.08–1.22	0.094	3.41	1.03–11.32	0.045	1.16	0.12–11.44	0.899	1.47	1.21–1.78	<0.001
	No (ref)												
Suicidal plan	Yes	4.78	10.7–21.32	0.040	1.78	0.29–10.89	0.532	1.89	0.91–18.26	0.584	1.97	1.59–2.42	<0.001
	No (ref)												
Loneliness experience	Yes	0.49	0.15–1.67	0.257	0.32	0.063–1.58	.161	2.87	0.43–19.25	0.275	1.13	0.95–1.35	0.152
	No (ref)												
Generalizedanxiety disorder	Mild	1.49	0.51–4.36	0.101	0	0	<0.001	0.33	0.08–1.43	0.175	1.37	1.14–1.64	<0.001
	Moderate	3.90	1.12–13.67		1.76	0.12–26.73		1.19	0.14–10.39		1.70	1.35–2.15	
	Severe	0.71	0.18–2.74		8.17	1.03–64.99		2.88	0.49–17.04		2.33	1.77–3.05	
	None (ref)												

OR, odds ratio; CI, confidence interval.

## Data Availability

All relevant data are included in the paper. Otherwise, the raw data analyzed during the current study are not publicly available as the owners provided their written consent only to the use of the data for the current study on IRB approval and for ethical reasons. The datasets used and/or analyzed in the present study are available from the corresponding author on reasonable request.
